# Editorial: Mitochondria at the nexus of metabolism, aging, and disease

**DOI:** 10.3389/fcell.2023.1356278

**Published:** 2024-01-11

**Authors:** Karthik Babu Mallilankaraman, Brian K. Kennedy, Vincenzo Sorrentino, Alessandro Luciani

**Affiliations:** ^1^ Department of Physiology, Yong Loo Lin School of Medicine, National University of Singapore, Singapore, Singapore; ^2^ Healthy Longevity Translational Research Programme, Yong Loo Lin School of Medicine, National University of Singapore, Singapore, Singapore; ^3^ Department of Biochemistry, Yong Loo Lin School of Medicine, National University of Singapore, Singapore, Singapore; ^4^ Mechanisms of Inherited Kidney Disorders Group, Institute of Physiology, University of Zurich, Zurich, Switzerland

**Keywords:** ageing, metabolism, mitochondrial dysfuncion, mitophagy, metabolic disease, dietary intervention, drug discovery, metabolic signaling

Mitochondria, the intracellular powerhouses responsible for converting nutrients into energy (in the form of ATP or heat), are highly dynamic, double-membraned organelles. They play a crucial role in a multitude of cellular functions, supporting energy metabolism and maintaining homeostasis. Recent exciting discoveries emphasize the importance of this ever-changing and functionally versatile organelle, particularly in terminally differentiated cell types heavily reliant on aerobic metabolism. Given the central role in metabolic and physiological homeostasis, dysregulation of mitochondria has been implicated not only in rare inherited diseases but also extended to common neurodegenerative and metabolic disorders, as well as cancer and age-related pathologies. Indeed, mitochondrial dysregulation is a hallmark of aging, perhaps in part explaining its role in such a wide range of pathologies. These findings underscore the pivotal role of mitochondria as a regulatory hub for homeostasis, making them an attractive and promising therapeutic target across a diverse spectrum of disease conditions.

The Research Topic, titled “Mitochondria at the Nexus of Metabolism, Aging, and Disease” in Frontiers in Cell and Developmental Biology, comprise a series of four articles unveiling recent breakthroughs in the cell biology of mitochondria, with relevance across health, disease, and therapeutic discovery. These articles delve into the intricate regulation of mitochondria by microenvironmental cues and shed light on challenges and unresolved questions addressing the biological functions of mitochondria in the context of health and disease.

The maintenance of intracellular calcium levels by mitochondria emerges as a key player in cellular bioenergetics and redox homeostasis. Over the past decade, research efforts have led to the identification of molecular components of the primary ion channel, the mitochondrial calcium uniporter (MCU), facilitating the permeation of Ca^2+^ into energized mitochondria to support bioenergetics. These findings have unveiled novel perspectives on the role of the mitochondrial calcium uniporter in age-related diseases, particularly in neurodegenerative and cardiovascular conditions. In their review, Walters and Usachev delved into the molecular components and mechanisms of mitochondrial calcium cycling in neuronal cells, focusing on neuronal mitochondrial calcium homeostasis. The review explored the disruptions in this signaling mechanism and their contribution to various disease states, providing detailed insights into both Ca^2+^ uptake and release mechanisms. The authors also discussed how components of the mitochondrial calcium machinery could be potential targets in neurodegenerative diseases.

The article by Garcia et al. provided a thorough investigation into the intricate role of MCU in cell proliferation and tumorigenesis. Using *in vitro* and *in vivo* models, they demonstrated that cancer cells sustain themselves by finely tuning the expression of MCU to enhance mitochondrial calcium uptake. Notably, the authors uncovered a previously unknown link between augmented MCU-mediated mitochondrial Ca^2+^ uptake, abnormalities in anaplerotic glucose (synthesis of intermediates necessary for maintaining glucose levels), and glutamine metabolism. Moreover, they confirmed that the deletion of MCU reduces anabolic programs for growth and cell proliferation in both cell- and animal (tumor xenografts)-based model systems. Mechanistically, the loss of MCU function appears to elevate glycolysis and glutaminolysis, sensitizing cell cycle progression to limitations in glucose and glutamine, and causing changes in agonist-induced cytoplasmic Ca^2+^ signals. Further insights into this signaling connection might ultimately lead to novel and effective treatments for diseases with large unmet needs, transforming our ability to regulate health and homeostasis.

Beyond the maintenance of physiological levels of intracellular calcium, mitochondria play a pivotal role in regulating other ions critical for homeostasis. Over the past decade, there has been a bourgeoning discovery of numerous ion channels, transporters, exchangers and solute carriers within mitochondria. The functional activation of many of these components necessitates post translational modifications (PTMs), and the disruptions in these PTMs contribute to the development of several (age-related) metabolic diseases. In a comprehensive review, Kadam et al. highlighted the role of mitochondrial ion channels and transporters in the context of PTMs, shedding new light on their contribution to functional activation and physiological homeostasis. The authors provided a critical analysis of disease-induced changes in these PTMs and discussed their role in regulating protein quality control. A molecular understanding of PTMs and their impact on the quality control mechanisms of mitochondrial channels and transporters can offer valuable insights for designing innovative therapeutic approaches for diseases driven by mitochondrial dysfunction. To advance our knowledge, further research efforts should focus on investigating the structural conformations governing the functioning states of these mitochondrial channels and transporters, understanding their transport mechanisms, and identifying functional interactors.

The mitochondrion serves as a command-and-control center for amino acid metabolism, where its reliance on mitochondrial enzymes closely intertwines with mitochondrial function. Perturbations in amino acid concentrations are evident in primary mitochondrial diseases and disorders characterized by mitochondrial dysfunction. In their review, Li and Hoppe presented a comprehensive summary of the current understanding of the molecular mechanisms governing the intricate interplay between amino acid metabolism and two pivotal energy-producing processes within mitochondria—the tricarboxylic acid (TCA) cycle and the respiratory chain. Furthermore, the authors explored existing evidence, particularly in non-mammalian and rodent model systems, highlighting how mitochondria contribute to the regulation of nutrient-sensing and geroprotective signaling pathways. These pathways, when activated, promote lifespan in flies, worms and in mice through controlled diet compositions and timing of food intake. There is great potential to witness these discoveries translated into clinical applications, utilizing a personalized food-as-medicine approach in the near future.

As we conclude this editorial piece, our heartfelt gratitude goes out to all the authors and referees for their invaluable contributions to this timely and up-to-date Research Topic. We anticipate that the Research Topic of articles will not only stimulate further research but also contribute to a deeper understanding of the biological functions of mitochondria in physiology, human disease, the aging process, age-related pathologies, and the potential impact of dietary and pharmacological interventions.

